# Conjunctival Intraepithelial Neoplasia With IgG and Complement Deposition: Spurious Association or Biologically Explainable?

**DOI:** 10.7759/cureus.103709

**Published:** 2026-02-16

**Authors:** Daniel D Zhang, Deepak Raja, Wang L Cheung, Curtis E Margo

**Affiliations:** 1 Ophthalmology, University of Central Florida College of Medicine, Orlando, USA; 2 Ophthalmology, Remagin Ophthalmology and Oculofacial Plastic Surgery Center, Orlando, USA; 3 Pathology, Orlando Health, Orlando, USA; 4 Ophthalmology, Pathology, and Cell Biology, University of South Florida Morsani College of Medicine, Tampa, USA

**Keywords:** complement c3, corneal intraepithelial neoplasia, direct immunofluorescence, igg, ocular cicatricial pemphigoid

## Abstract

Conjunctival intraepithelial squamous neoplasia (CIN) is a premalignant ocular surface lesion typically diagnosed by clinical features and confirmed with routine histopathologic examination, rather than immunofluorescence studies. Direct immunofluorescence (DIF) of conjunctival tissue is most commonly used to support the diagnosis of autoimmune blistering diseases, particularly mucous membrane pemphigoid, by identifying immunoreactant deposition along the basement membrane zone (BMZ). However, such findings are not expected in CIN and may complicate interpretation.

We report a case of CIN with unexpected positive DIF findings of IgG and C3 in the BMZ. A 63-year-old man with a clinically apparent conjunctival lesion underwent biopsy, which confirmed CIN on routine histopathology. A portion of the specimen was inadvertently submitted for DIF, revealing linear deposition of IgG and C3 along the conjunctival BMZ. The patient had no clinical history or examination findings suggestive of mucous membrane pemphigoid, other autoimmune blistering disease, or systemic inflammatory disorder that could account for these immunoreactant deposits. This association suggests nonspecific deposition of immunoglobulins and complement in the setting of conjunctival neoplasia, a phenomenon rarely reported in the literature. This case highlights the potential for false-positive DIF results in conjunctival lesions and emphasizes the importance of submitting a concomitant biopsy for routine light microscopy when ordering DIF in patients with suspected mucous membrane pemphigoid to ensure accurate diagnosis.

## Introduction

Conjunctival intraepithelial neoplasia (CIN) is the most common type of ocular surface neoplasm and is characterized by squamous intraepithelial dysplasia of varying severity [1-3]. Clinically, conjunctival intraepithelial neoplasia (CIN) typically presents as a fleshy, minimally elevated lesion involving the limbal conjunctiva, often with extension onto the adjacent corneal epithelium. Based on clinical appearance alone, CIN may be difficult to distinguish from benign, inflammatory, or degenerative disorders of the conjunctiva [4]. Diagnosis often involves histopathologic confirmation but rarely requires immunohistochemical studies [5].

Direct immunofluorescence (DIF) of conjunctival tissue, on the other hand, is obtained primarily in the context of a limited number of diseases, particularly those suspected of immune-mediated injury, most commonly cicatrizing conjunctivitis due to mucous membrane pemphigoid (MMP) [6]. DIF may demonstrate linear deposition of immunoglobulins and complement along the epithelial basement membrane zone (BMZ). The BMZ is a specialized extracellular matrix interface between the conjunctival epithelium and underlying stroma that is a key target in autoimmune subepithelial blistering disorders.

Immunoreactant deposition at the BMZ can provide supportive diagnostic evidence when interpreted in conjunction with clinical findings and routine histopathology. However, DIF is not fully sensitive for MMP, and negative results do not exclude the diagnosis in patients with typical clinical features [6,7]. Moreover, positive immunoreactant deposition along the conjunctival BMZ is not disease-specific and has been reported in a variety of other settings, including bullous pemphigoid, linear IgA disease, paraneoplastic syndromes, and drug-induced conjunctival cicatrization [8]. The presence of immunoreactants has also not been extensively studied in diseases other than autoimmune. Therefore, direct DIF findings must be interpreted within the appropriate clinical and histopathologic context.

We describe a patient with CIN whose biopsy was inadvertently submitted for DIF and demonstrated linear deposition of immunoglobulin G (IgG) and complement component C3 (C3) positivity along the conjunctival BMZ. This positive DIF finding was unexpected in CIN, since it is usually associated with autoimmune bullous or cicatrizing disorder of skin or mucous membrane [9,10]. This laboratory result raised questions regarding the specificity of conjunctival DIF and how often immunoreactant deposition along the BMZ may occur outside the context of autoimmune disease.

## Case presentation

**Figure 1:**
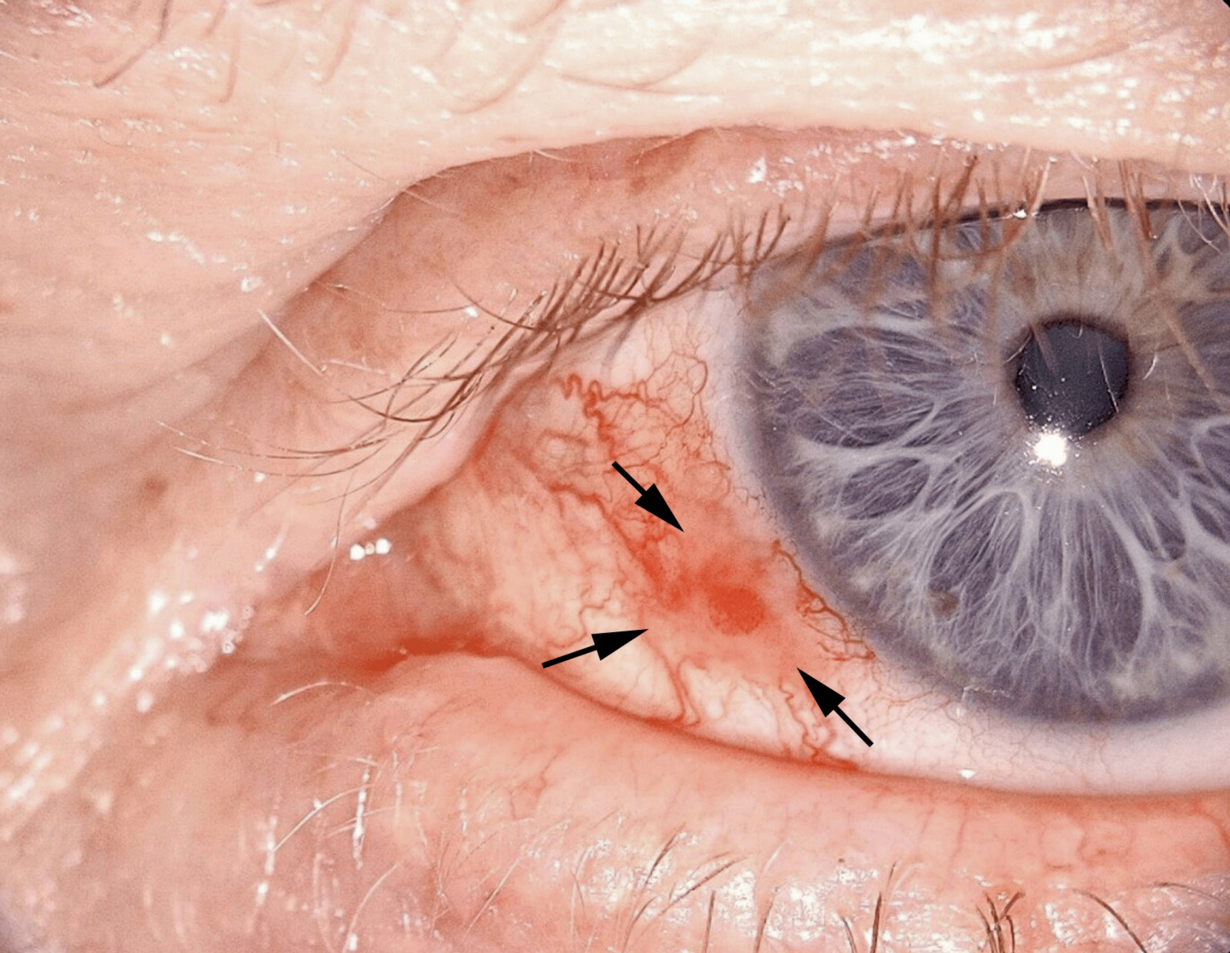
Clinical Appearance of the Conjunctival Lesion A vascularized, slightly elevated gelatinous mass is present from 7 to 9 o’clock, contiguous with the limbus. The lesion had been noticed for two months. The arrows indicate the lesion and outline the in situ tumor.

A 63-year-old man presented with a lesion on his left eye for 2 months. The patient has no prior history of malignancy, autoimmune disease, or relevant family history. His general medical and ophthalmic histories were non-contributory. His ocular examination was remarkable for an inflamed, slightly elevated gelatinous mass of the left nasal conjunctiva contiguous with the limbus and extending from 7 to 9 o’clock (Figure 1). No other abnormalities of the conjunctiva were noted in either eye, including symblepharon.

**Figure 2:**
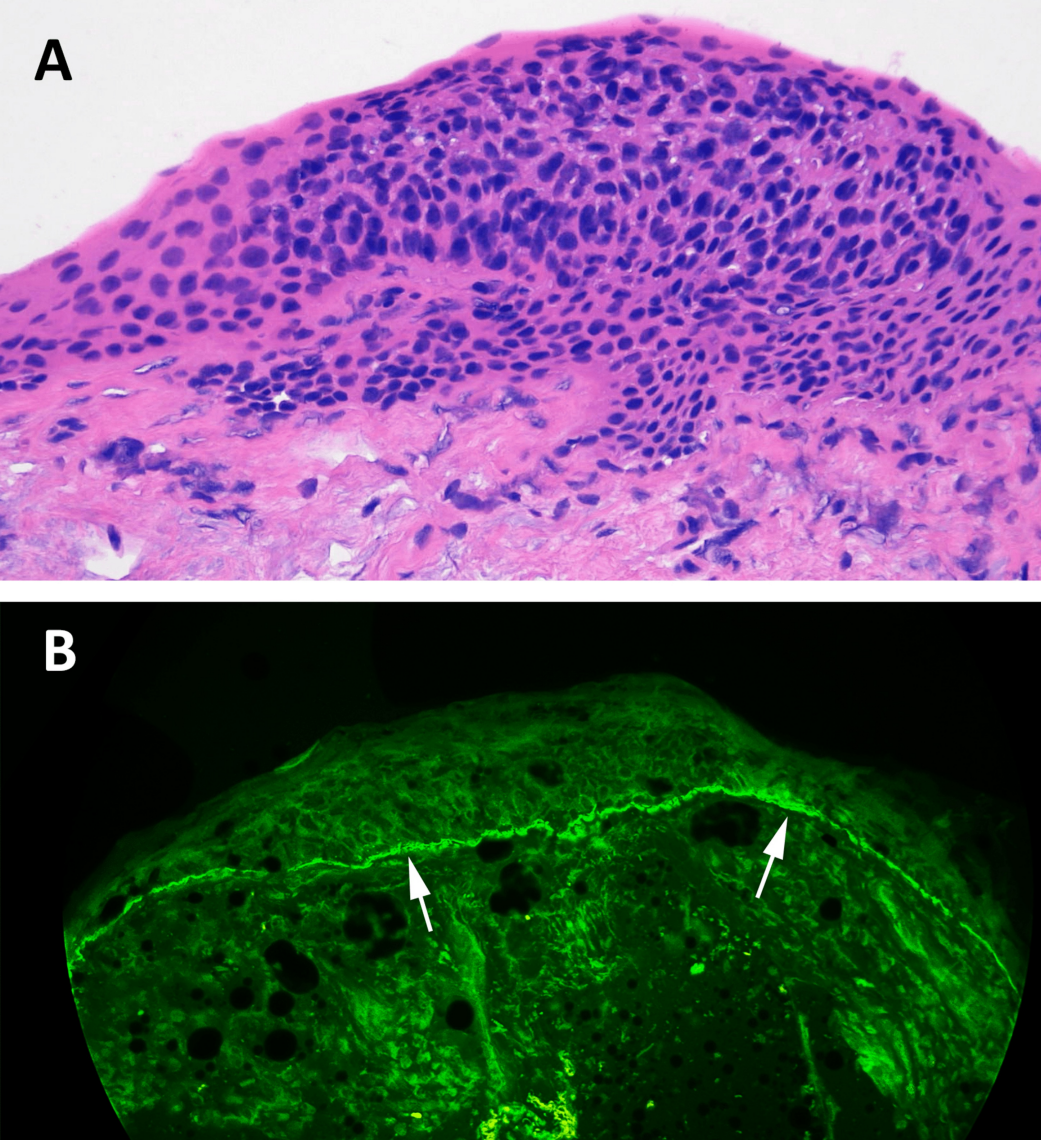
Histopathology of the Lesion: Hematoxylin and Eosin (H&E) and Direct Immunofluorescence (DIF) Stains (A) Hematoxylin and eosin–stained section; magnification 300×. In this slightly oblique-cut section, the conjunctival epithelium displays disorganized cellular maturation. Individual cells have increased nuclear-to-cytoplasmic ratios and dense nuclear chromatin. There is full-thickness dysplasia. (B) Direct immunofluorescence for IgG shows strong positivity along the basement membrane zone of the conjunctiva.

The mass was excised without difficulty. Half the specimen was inadvertently submitted for DIF. Histologically, the biopsy displayed full-thickness intraepithelial cellular atypia and disordered cellular maturation (Figure 2A). On DIF, there was linear deposition of IgG (Figure 2B) and C3 along the epithelial BMZ.

The diagnosis of CIN, carcinoma in situ, was reported along with the results of DIF. Follow-up clinical evaluation failed to detect any evidence of cutaneous or mucous membrane disease. There was no recent or past history of systemic cancer that might indicate a paraneoplastic condition. The patient has been followed for 3 months without any new relevant findings.

## Discussion

Conjunctival intraepithelial neoplasia (CIN) represents a localized, premalignant proliferation of dysplastic squamous epithelium and is considered part of the spectrum of ocular surface squamous neoplasia [1-3]. Diagnosis is typically established through routine histopathologic examination, and ancillary immunohistochemical or immunofluorescence studies are not generally required. In contrast, direct immunofluorescence (DIF) of conjunctival tissue is typically employed in the evaluation of suspected immune-mediated cicatrizing conjunctivitis, particularly mucous membrane pemphigoid (MMP) [9,10]. In this clinical context, it is an important diagnostic adjunct. The number of false-positive cases of DIF reported in the literature is small, which may reflect the fact that most DIF controls are normal conjunctiva. In our case, the CIN was inadvertently submitted for DIF and found to be positive. The possibility of false-positive DIF for autoimmune disease may be greater than believed because other forms of conjunctival pathology have not been thoroughly studied.

In ocular MMP, DIF classically demonstrates linear deposition of immunoglobulins (most often IgG and/or IgA) and complement, frequently C3, along the epithelial BMZ [9,10]. Although DIF is widely regarded as a key component of the diagnostic workup, its sensitivity is imperfect and depends on multiple factors, including biopsy site selection, disease activity, and tissue handling [9]. Moreover, DIF findings must be interpreted in conjunction with clinical features and routine histopathology, as linear BMZ immunoreactant deposition is not specific to MMP and may be observed in other autoimmune blistering diseases [10]. The frequency of DIF-positive reactivity has not been thoroughly investigated in CIN.

The association of CIN and DIF positivity for IgG and C3 along the conjunctival BMZ raises several diagnostic considerations. Specimen mislabeling was initially considered but could be excluded given that the same morphologic changes were found in both tissues. It is also considered that the patient had two concurrent and unrelated disorders (e.g., CIN and mucous membrane pemphigoid). However, given the absence of a history of previous cutaneous or mucous membrane disease and the lack of clinically visible lesions, this possibility was considered unlikely. Although testing for circulating autoantibodies to BP180 was considered, it was deferred, as even positive results would not have altered clinical management.

Another possibility is that IgG and C3 deposition in the conjunctival epithelial BMZ could be a sign of occult malignancy, serving as a subclinical manifestation of a pemphigoid-like paraneoplastic disorder [11]. While this possibility exists, the patient had no clinical or historical features suggestive of systemic malignancy and opted for periodic medical follow-up rather than empirical laboratory and imaging studies. The absence of systemic symptoms or disease progression during follow-up makes this explanation less likely in the present case.

Prior reports have documented the coexistence of CIN and MMP. Choi and colleagues reported two cases of CIN arising in patients with established mucous membrane pemphigoid, both of which demonstrated BMZ immunoreactant positivity for IgG, IgA, and fibrinogen on DIF [12]. These cases appear to represent the coincidental occurrence of two uncommon and unrelated disorders, rather than a shared pathogenic mechanism. As noted above, a large series of patients with CIN has not been studied for their immunoreactivity. 

Grossniklaus and colleagues described a case of sebaceous carcinoma of the eyelid that clinically mimicked cicatricial pemphigoid and exhibited linear IgA and C3 deposition at the cutaneous BMZ [13]. Histopathology, however, documented an invasive adnexal carcinoma, and the patient had no other evidence of cicatricial disease. The association was thought to be anomalous or represent a false-positive laboratory result, which is admittedly an unsatisfying explanation.

Taken together, the findings in the present case raise the possibility that IgG and C3 deposition in the BMZ exists in subclinical form more often than currently appreciated. Do autoantibodies to target antigens in the BMZ, such as BP180, BP230, laminin 5, or β4 integrin, occur in individuals without causing overt disease? Wang and associates found anti-BP180 autoantibodies in 23 of 48 patients (48%) with Alzheimer’s disease (without clinical bullous pemphigoid) compared with only four of 50 control patients (8%) [14]. These serology studies (particularly in the control group) suggest that it is possible for persons to harbor autoantibodies that could explain the rare and otherwise inexplicable association of CIN with linear deposition of IgG and C3 in the conjunctival BMZ. Rare false-positive DIF results have also been described in various inflammatory dermatoses and, by exclusion, were considered deposits of non-specific immunoreactants [15]. It is thus possible that DIF-positivity in other conjunctival pathologies (such as CIN) is more common than previously believed because they have not been systematically studied. 

## Conclusions

This case describes a CIN associated with unexpected linear IgG and C3 deposition along the conjunctival basement membrane zone on DIF. Although such immunopathologic findings are associated with MMP, the absence of clinical or histopathologic features of cicatrizing autoimmune disease in this patient suggests that BMZ immunoreactant deposition may occur in non-autoimmune conjunctival pathology, including CIN. 

This observation highlights the limited specificity of conjunctival DIF in the evaluation of suspected ocular cicatricial disease. DIF findings, while valuable, are not disease-specific and must be interpreted in conjunction with clinical and histopathologic findings. Reliance on DIF alone may lead to misdiagnosis and unnecessary systemic evaluation or treatment.

Accordingly, when DIF is obtained for suspected MMP, submission of a concomitant biopsy for light microscopy is strongly recommended. Awareness that ocular surface neoplasia may be associated with positive immunofluorescence findings may help clinicians avoid unnecessary diagnostic workup and ensure appropriate patient management. Further study is warranted to better define the frequency and mechanisms of nonspecific BMZ immunoreactant deposition in conjunctival disease.
